# Complex oscillatory redox dynamics with signaling potential at the edge between normal and pathological mitochondrial function

**DOI:** 10.3389/fphys.2014.00257

**Published:** 2014-07-08

**Authors:** Jackelyn M. Kembro, Sonia Cortassa, Miguel A. Aon

**Affiliations:** ^1^Facultad de Ciencias Exactas, Físicas y Naturales, Instituto de Investigaciones Biológicas y Tecnológicas (Consejo Nacional de Investigaciones Científicas y Técnicas-UNC) and Instituto de Ciencia y Tecnología de los Alimentos, Universidad Nacional de CórdobaCórdoba, Argentina; ^2^Division of Cardiology, Department of Medicine, Johns Hopkins University School of MedicineBaltimore, MD, USA

**Keywords:** ROS signaling, mitochondrial energetic/redox, complex oscillations, Hopf bifurcations, physiological and pathophysiological behavior, redox environment

## Abstract

The time-keeping properties bestowed by oscillatory behavior on functional rhythms represent an evolutionarily conserved trait in living systems. Mitochondrial networks function as timekeepers maximizing energetic output while tuning reactive oxygen species (ROS) within physiological levels compatible with signaling. In this work, we explore the potential for timekeeping functions dependent on mitochondrial dynamics with the validated two-compartment mitochondrial energetic-redox (ME-R) computational model, that takes into account (a) four main redox couples [NADH, NADPH, GSH, Trx(SH)_2_], (b) scavenging systems (glutathione, thioredoxin, SOD, catalase) distributed in matrix and extra-matrix compartments, and (c) transport of ROS species between them. Herein, we describe that the ME-R model can exhibit highly complex oscillatory dynamics in energetic/redox variables and ROS species, consisting of at least five frequencies with modulated amplitudes and period according to power spectral analysis. By stability analysis we describe that the extent of steady state—as against complex oscillatory behavior—was dependent upon the abundance of Mn and Cu, Zn SODs, and their interplay with ROS production in the respiratory chain. Large parametric regions corresponding to oscillatory dynamics of increasingly complex waveforms were obtained at low Cu, Zn SOD concentration as a function of Mn SOD. This oscillatory domain was greatly reduced at higher levels of Cu, Zn SOD. Interestingly, the realm of complex oscillations was located at the edge between normal and pathological mitochondrial energetic behavior, and was characterized by oxidative stress. We conclude that complex oscillatory dynamics could represent a frequency- and amplitude-modulated H_2_O_2_ signaling mechanism that arises under intense oxidative stress. By modulating SOD, cells could have evolved an adaptive compromise between relative constancy and the flexibility required under stressful redox/energetic conditions.

## Introduction

Poised at the convergence of most catabolic and anabolic pathways, mitochondria are at the center of heterotrophic aerobic life, representing a hub in the cellular metabolic network (Aon et al., 2007a; Aon and Cortassa, 2012; Cortassa and Aon, 2013; Kembro et al., 2014). The energetic functions performed by mitochondria face the unavoidable redox hurdle of handling huge amounts of O_2_ while keeping their own as well as the cellular redox environment. Mitochondria produce ~85–90% of cellular reactive oxygen species (ROS) (Chance et al., 1979; Shigenaga et al., 1994; Balaban et al., 2005), while supplying the bulk of ATP demanded by the organs in the human body. The heart consumes proportionately most of the O_2_ on a specific basis with respect to the whole human body (Rolfe and Brown, 1997) thereby becoming especially vulnerable to oxidative damage. Although myocardial function declining with age, as well as the ability of the heart to tolerate stress (Lakatta and Sollott, 2002), are not understood mechanistically, mitochondrial dysfunction, oxidative stress and the accumulation of oxidant-induced damage are major contributing factors (Fannin et al., 1999; Suh et al., 2003; Judge et al., 2005a,b).

Originally considered an unavoidable and dangerous byproduct of oxidative phosphorylation (OxPhos), more recently we have become aware of the crucial role played by ROS signaling in key cellular functions. If under control, H_2_O_2_ becomes recognized as a specific signaling molecule, but beyond physiological limits it can readily become damaging. Under pathophysiological conditions, excessive ROS levels can occur due to either alterations in production, overwhelming of antioxidant defenses, or both (Aon et al., 2003, 2007a; Jones and Go, 2010). However, levels compatible with signaling are attained when production and scavenging of ROS are balanced within mitochondria and cells.

The redox environment (RE) determines the relationship between mitochondrial respiration and ROS. At maximal respiratory rate, mitochondrial ROS emission trends to a minimum and exhibits a clear dependence on the RE, from ~400 to 900 mV·mM in state 4 respiration and ~500 to 300 mV·mM in state 3 respiration (Cortassa et al., 2014). The dependence of ROS on mitochondrial respiration involves two terms: production and emission; whereas the former depends on respiration (i.e., the rate of electron transport through the respiratory chain) the latter relies on the balance between the production and scavenging roles. The ROS scavenging capacity is tightly linked to the redox-energetic status of mitochondria. NAD(P)H is the main electron donor to the antioxidant systems, but its generation depends on NADH, which exerts a dual redox and energetic role through transhydrogenase and complex I acceptors, respectively.

Recent data highlight the dominant role exerted by the glutathione (GSH) and thioredoxin (Trx) scavenging systems on H_2_O_2_ emission dynamics from mitochondria (Stanley et al., 2011; Kembro et al., 2013), especially under state 3 respiration when the energetic output is maximal (Aon et al., 2012; Cortassa et al., 2014). These data suggest that the GSH/Trx systems continuously scavenge ROS produced in the respiratory chain, thereby demonstrating that the antioxidant systems play a determinant task in the dynamics of H_2_O_2_ release by mitochondria. In this scenario, the emerging role of mitochondria as signaling organelles and ROS as signaling molecules increases the importance of understanding the dynamics of ROS emission and its role in normal as well as stress conditions. Mitochondria were shown experimentally and theoretically to be autonomous oscillators (Aon et al., 2003, 2008b; Cortassa et al., 2004; Kurz et al., 2010a; Qu, 2013) thus potentially representing a frequency- and amplitude-modulated signaling mechanism that could connect energetics to ROS-activated signaling pathways, including those responsible for regulating gene transcription (Morel and Barouki, 1999; Misra et al., 2003; Aon et al., 2006, 2007a, 2008a).

Duplication of antioxidant defense systems in multiple compartments can be an efficient salvage mechanism in response to oxidative bursts, and as a modulator of ROS dynamics. Superoxide dismutase (SOD) represents a relevant example of duplicated ROS scavenging systems localized in distinct compartments. Mammals have three isoforms of SOD present in the extracellular Cu, Zn SOD (SOD3), cytoplasmic Cu, Zn SOD (SOD1) and the mitochondrial Mn SOD (SOD2), compartments. Together they constitute the major antioxidant defense systems in charge of safely modulating O^−^_2_. Exposure to oxidants can act as a signal to increase the activities and expression of antioxidant enzymes (Rodriguez et al., 2004), and as a result an increase in antioxidant enzyme activity with age is expected to help protect tissues from oxidative stress (Judge et al., 2005a).

Compartmentalization is relevant in the control of ROS levels and the redox environment (Kembro et al., 2013), but its role in the dynamics of mitochondrial signaling is unknown. Although each subcellular compartment exhibits its own dynamics, the interdependence of their permeant redox status is mediated by the exchange of redox species (e.g., GSH, ROS). A previous version of our computational model of mitochondrial function showed frequency- and amplitude-modulated oscillations (Cortassa et al., 2004). These autonomous oscillations could span several orders of magnitude (milliseconds to several hours) by simply changing one parameter, e.g., the SOD concentration in the extra-mitochondrial compartment (Cortassa et al., 2004; Aon et al., 2006, 2008b). However, unexplained in this early model formulation was the impact exerted by the duplication of SODs in mitochondrial matrix and cytoplasm, and the exchange rates of O^−^_2_, H_2_O_2_, and GSH between compartments. Consequently, in the present work we investigate the role played by the compartmentalization of SODs on the oscillatory dynamics of H_2_O_2_. We focus on SOD1 and SOD2 because of their demonstrated critical role in cell physiology, as well as whole organism survival, lifespan, and disease states (Antila and Westermarck, 1989; Tribble et al., 1997; Sun and Tower, 1999; Craven et al., 2001a,b; Melov et al., 2001; DeRubertis et al., 2004; Sun et al., 2004; Kowluru et al., 2006a,b; Lu et al., 2009; Massaad et al., 2009a,b; Usui et al., 2009, 2011; Fukai and Ushio-Fukai, 2011).

## Materials and methods

### Computational model

A two-compartment mitochondrial energetic-redox (ME-R) model (Kembro et al., 2013) was utilized to assess the influence of ROS production and antioxidant systems on the period, amplitude and waveform of mitochondrial oscillations. The ME-R model incorporates four main redox couples [NADH/NAD^+^, NADPH/NADP^+^, GSH/GSSG, Trx(SH)2/TrxSS]. Superoxide dismutases (SOD) and other scavenging systems—glutathione, thioredoxin, catalase—distributed in mitochondrial matrix and extra-matrix compartments, and transport between compartments of ROS species (superoxide: O^−^_2_, hydrogen peroxide: H_2_O_2_), and GSH are also taken into account.

The model also accounts for respiratory flux from substrates of complex I and complex II, pH effects on equilibrium constants and enzyme activity, ion dynamics (Wei et al., 2011), the shunt of electrons from the respiratory chain toward the generation of O^−^_2_ (Shunt), and a ROS-activated anion efflux pathway across the inner membrane (Cortassa et al., 2004). Synthesis of NADPH from NADP^+^ and NADH via isocitrate dehydrogenase 2 (IDH_2_) and transhydrogenase (THD), respectively, are also included in the ME-R model.

**Figure 1:**
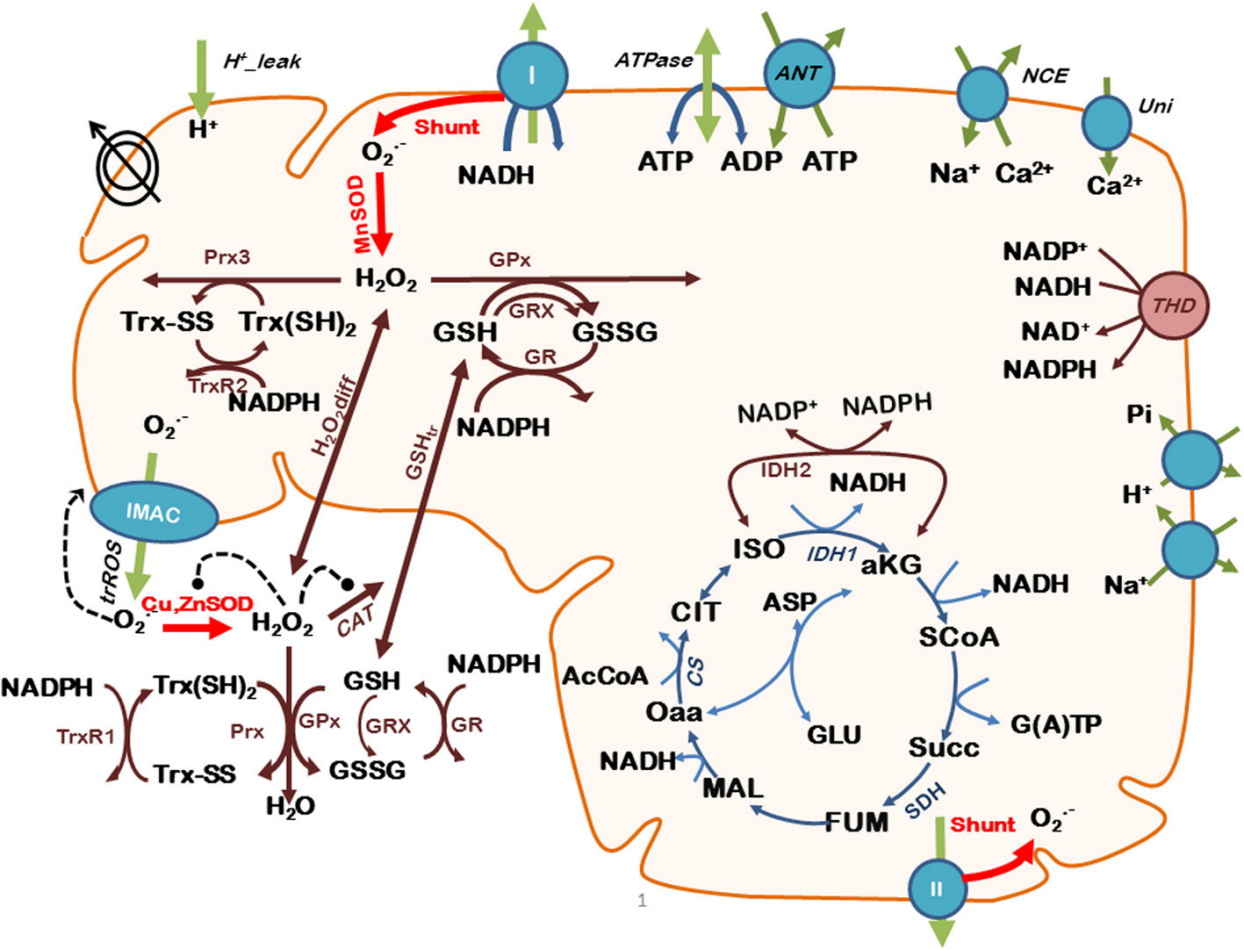
**Scheme of the two-compartment ME-R model accounting for mitochondrial energetic and redox processes, their interactions, and transport between compartments**. The model takes into account oxidative phosphorylation (OxPhos) and matrix-based processes in mitochondria as well as in the extra-mitochondrial compartment. In addition to energy metabolism and ion transport (H^+^, Ca^2+^, Na^+^, Pi), the model accounts for O^−^_2_ being produced in the mitochondrial electron transport chain from both complex I- and complex II-derived electron transport. O^−^_2_ may be dismutated to H_2_O_2_ by matrix-localized superoxide dismutase (MnSOD) or be transported to the extra-mitochondrial compartment through the inner membrane anion channel (IMAC), where it will be scavenged by Cu,ZnSOD. H_2_O_2_ can either diffuse from the matrix or be scavenged by the large capacity glutathione (GSH) and thioredoxin (Trx) systems, or by catalase (CAT) in the extra-mitochondrial compartment. Glutaredoxin (Grx) accounts for the recovery of glutathionylated proteins in the matrix. *Key to symbols*: Concentric circles with an arrow across represent the ΔΨ_m_. Dotted arrows indicate regulatory interactions either positive (*arrowhead*) or negative (• **--**). “Shunt” indicates the fraction of electrons from respiration diverging toward O^−^_2_. The red arrows highlight the model state variables (SODs and Shunt) that will be evaluated with respect to their impact on mitochondrial oscillations. Modified from Kembro et al. (2013).

The scheme for the integrated model is shown in Figure 1, and its complete description as well as parameterization is described elsewhere (Kembro et al., 2013).

### Model simulations

All studies were performed using the parametric setting with which the ME-R model was able to simulate different experimental situations (Kembro et al., 2013), with the exception of the concentrations of Mn SOD and Cu, Zn SOD, and Shunt values.

Numerical integration of model equations (ODE15s) was performed with MatCont 2.4 (Dhooge et al., 2008) in MATLAB 7.1, until steady-state solutions were obtained (i.e., when the magnitude of each time derivative was <10^−10^). Steady-state values of each state variable were then used as input for performing bifurcation and continuation analysis performed with MatCont 2.4 (Dhooge et al., 2008) in MATLAB 7.1. This software is used to determine the dependence of steady-state solution properties (type and stability) on model parameters. Eigenvalues characterizing the bifurcation properties of the ME-R model were also analyzed with MatCont 2.4. For stability analysis, the Shunt was utilized as the bifurcation parameter at fixed concentrations of mitochondrial superoxide dismutase (Mn SOD) and extra-mitochondrial superoxide dismutase (Cu,Zn SOD).

Time series analysis was performed on series with a duration of 1.6. 10^7^ ms obtained by numerical integration of model equations using absolute tolerance of 10^−14^ and relative tolerance of 10^−9^. The solutions were then evaluated according to Kierzenka and Shampine (2011) in MATLAB R2013a to obtain time series with constant sampling frequency at 1 ms. The system was simulated for an extended period of time (i.e., at least 2. 10^9^ ms) to ascertain the achievement of stationary time series. These time series were then analyzed by power spectral analysis using the Fast Fourier Transform (FFT) subroutine of Matlab. Due to the stationarity of the time series they were not preprocessed or filtered.

## Results

### Extra-mitochondrial cuznsod determines oscillatory mitochondrial dynamics at the edge between functional and pathological behavior

We investigated the dependence of the mitochondrial dynamic behavior (onset and extent of oscillatory behavior) as a function of three key model parameters (concentrations of Mn SOD and Cu, Zn SOD, and Shunt). Mitochondrial dynamics evolves toward a steady state (i.e., fixed point attractors) or oscillations (i.e., limit cycles) depending on the antioxidant capacity of the mitochondrial and extra-mitochondrial compartment via Mn SOD and Cu, Zn SOD, respectively, when Shunt (i.e., ROS production) is increased. We analyzed the appearance of three distinct mitochondrial states: (1) functional (i.e., highly reduced NADH, polarized membrane potential and minimum ROS release), (2) pathological (i.e., highly oxidized NADH, depolarized membrane potential and high ROS release), and (3) oscillatory (i.e., oscillations in main bioenergetic variables such as ROS release, membrane potential, tricarboxylic acid (TCA) cycle intermediates, and antioxidant systems).

**Figure 2:**
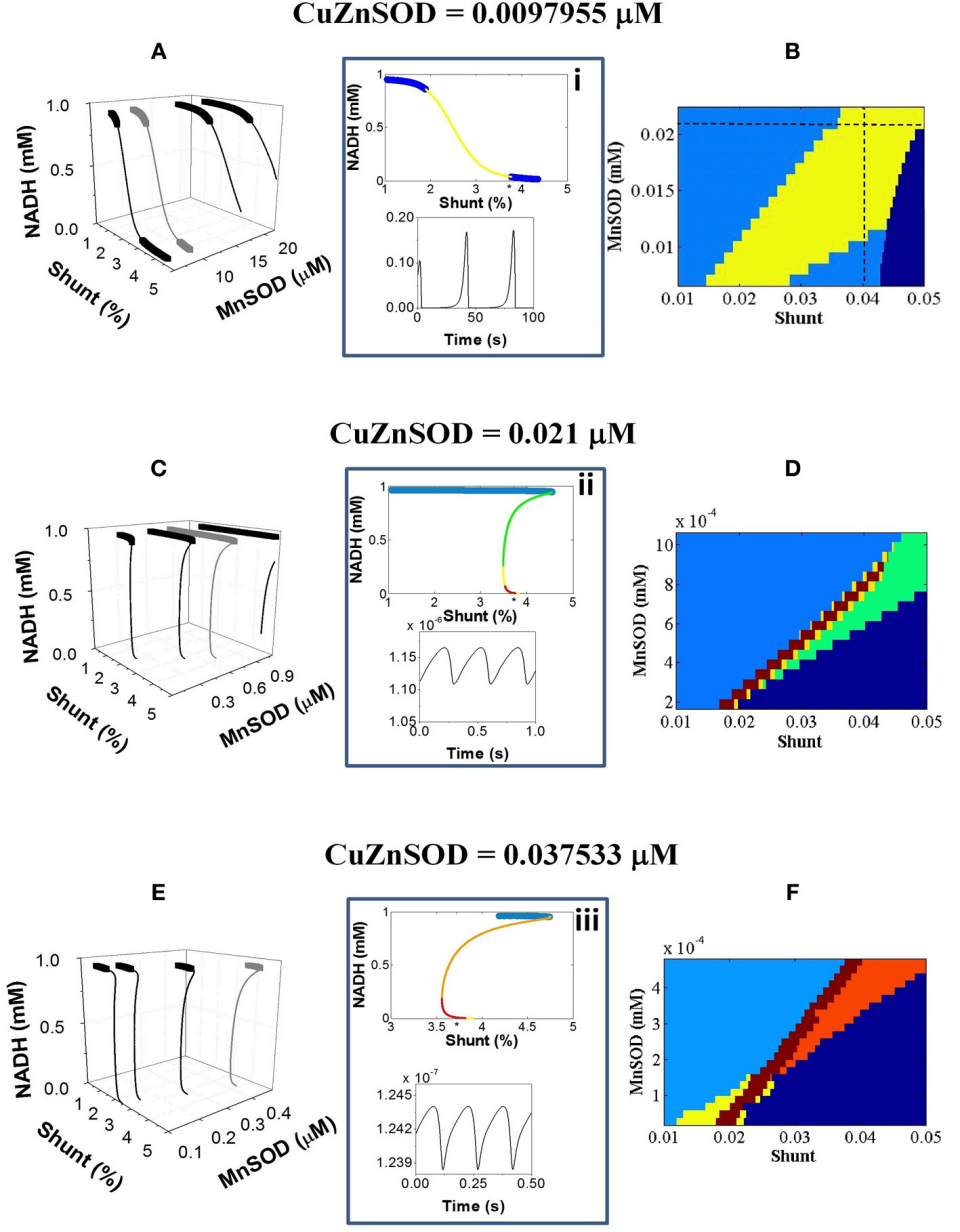
**NADH dynamic behavior as a function of mitochondrial ROS production and scavenging, at different antioxidant capacity of the extra-mitochondrial compartment**. The model behavior was analyzed by stability analysis as described in Methods. **(A,C,E)** Depicted are the bifurcation diagrams showing an upper branch of steady states in which NADH is predominantly reduced, and a lower branch in which NADH is mainly oxidized. Thick lines correspond to stable steady-state behavior whereas thin lines denote stable limit cycle (oscillatory) behavior and an unstable focus. Hopf bifurcations mark the transition from steady to oscillatory dynamics. Further analysis of the eigenvalues obtained in the stability analysis enabled a detailed description of the oscillatory region. Insets i–iii depict an example of eigenvalues for the bifurcation diagrams shown in gray in the panels **(A,C,E)** at the left. Information of the maximum eigenvalue observed for a given parametric combination is represented in the colored plots in the **(B,D,F)** panels at the right. In the stable region of the diagram denoted in light blue (normal behavior), all negative real values (i.e., stable steady states corresponding to fixed point attractors) were found whereas in the oscillatory region at least one positive real eigenvalue could be determined (i.e., sustained oscillations corresponding to a stable limit cycle). Green, yellow, orange and red colors code for 1, 2, 3, or 4 real positive eigenvalues, respectively. Dark blue indicates the pathological domain corresponding to non-functional (“dead”) mitochondria. The asterisk (^*^) on the *x-axis* of top panel from the middle insets (i–iii) corresponds to the Shunt value at which the time series represented at the bottom panel was obtained. In panels **(B,D,F)** the maximum number of positive eigenvalues found for each parametric combination is represented with the same color code used in the insets. The dotted lines in panel **(B)** correspond to the parametric combinations giving rise to complex oscillations shown in **Figure 5**.

Figure 2 depicts a more detailed exploration of mitochondrial redox (NADH) behavior as a function of SODs and Shunt using stability analysis. An extensive oscillatory region delimiting functional from pathological domains of mitochondrial behavior appears as a function of increasing ROS production, i.e., higher Shunt (Figure 2B). This oscillatory region becomes more confined as the antioxidant capacity of Cu, Zn SOD in the extra-mitochondrial compartment is enhanced (Figures 2D,F).

The bifurcation diagrams evolve from smoother to steeper S-shapes depending on the concentration of Cu, Zn SOD (Figures 2A,C,E). Unlike the typical S-shape behavior exhibited by bistable systems, the transition between the upper (reduced) and lower (oxidized) branches of NADH states in the two-compartment ME-R model is not done abruptly at limit points (Aon and Cortassa, 1997; Cortassa et al., 2004). In contrast, the thin line connecting upper and lower branches of steady states in the bifurcation diagrams from Figure 2 exhibits both an unstable focus and a stable limit cycle (see insets i–iii from Figure 2). According to the stability analysis, the limit cycles appear after Hopf bifurcations (HBs) exhibiting 2 and up to 4 positive eigenvalues corresponding to the real component of the complex imaginary numbers characterizing HBs, i.e., the higher the Cu, Zn SOD concentration the higher the number of positive eigenvalues (Figures 2B,D,F). A positive eigenvalue implies sustained oscillations whereas a higher number of them suggest different types of oscillatory behavior (see **Figure 5** below).

Combinations of higher Mn SOD and/or Cu, Zn SOD concentrations bestow a higher tolerance to ROS produced before the system transitions toward oscillations or steady (but depolarized) states (Figure 2). Low values in either class of SOD can be reciprocally compensated by higher values of the other thus preserving conditions compatible with life under oxidative stress (Figures 2B,D,F). Consequently, it appears that both SODs can compensate each other to maintain functionally compatible dynamic behavior. Qualitatively, the dynamic behavior of the model agrees with experimental evidence showing that either increasing the concentration of ROS scavengers, or inhibiting respiration to decrease mitochondrial ROS production, inhibits oscillations in ΔΨ_m_ by stabilizing the polarized steady state, or by distancing the mitochondrial network from criticality, i.e., preventing ROS accumulation to the critical threshold (Aon et al., 2003, 2004; Cortassa et al., 2004).

### Complex oscillatory behavior at the edge of normal and pathological mitochondrial behavior

**Figure 3:**
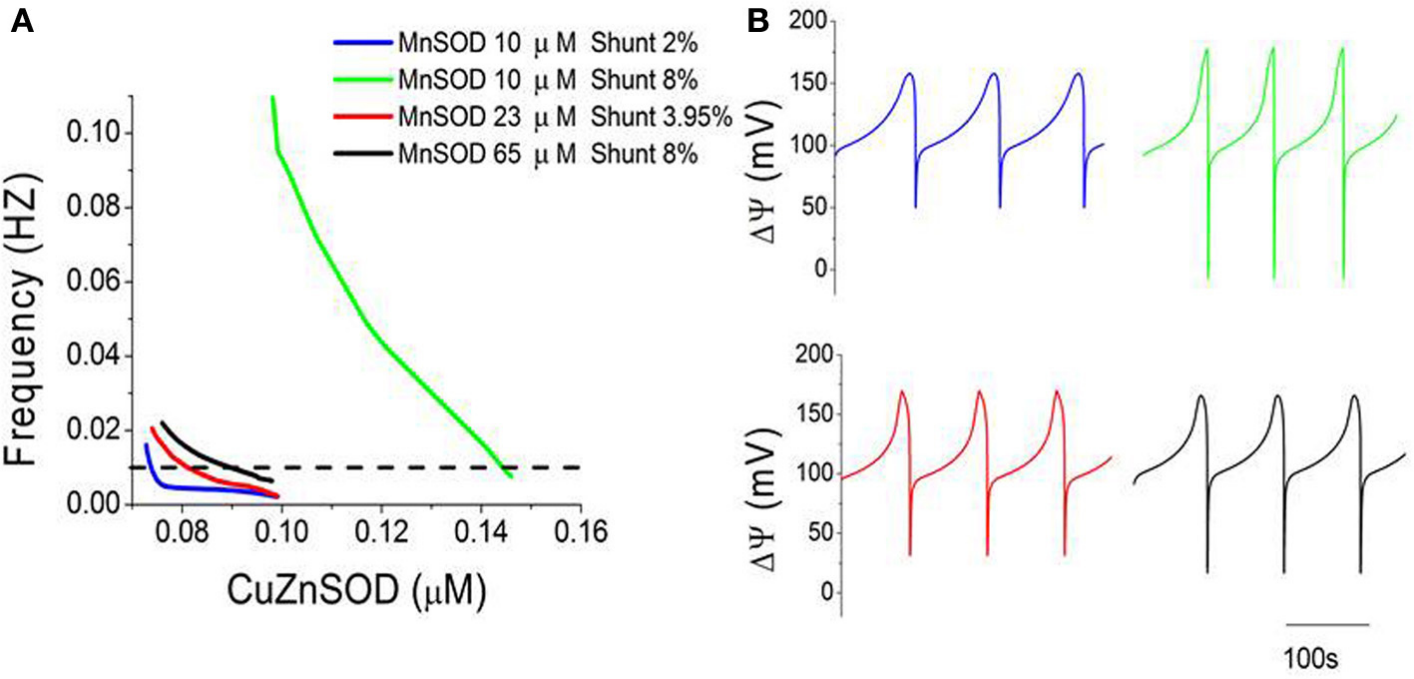
**Three-way modulation of the oscillations' frequency in mitochondrial membrane potential. (A)** The frequency (1/period) of mitochondrial oscillations as a function of increasing concentrations of CuZnSOD at four different combinations of MnSOD and Shunt. Notice that the oscillator may attain the same frequency (0.01 Hz, or 100 s period) with different combinations of the three parameters (MnSOD, CuZnSOD, and shunt) as indicated by the dotted line. **(B)** Displayed are the time series corresponding to the four parametric combinations shown in **(A)** at a frequency of 0.01 Hz.

**Figure 4:**
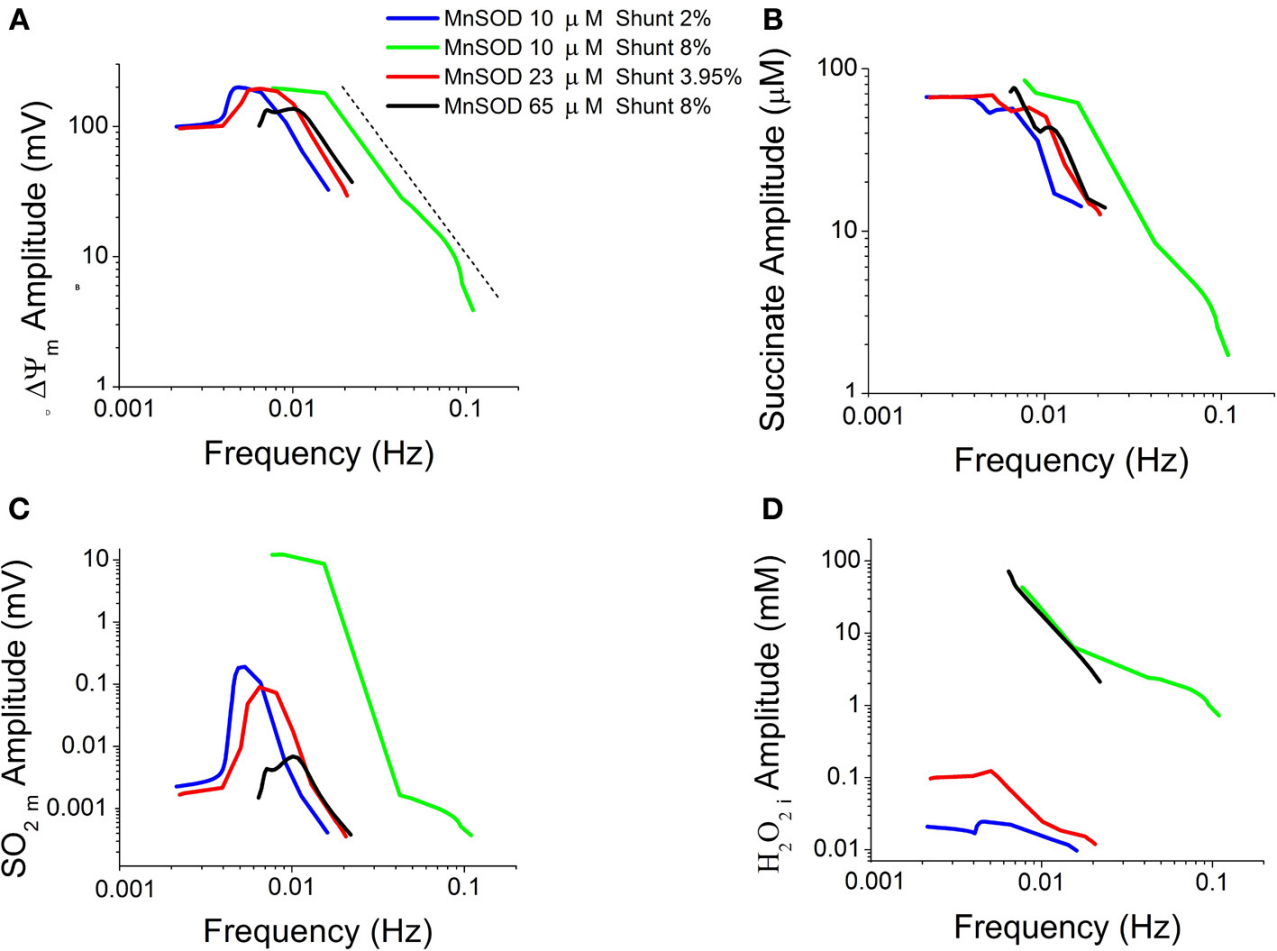
**Double-log plot of oscillation amplitude vs. frequency for different time series**. Characterization of oscillatory behavior in energetic (mitochondrial membrane potential, ΔΨ_m_, succinate, Succ) and redox (mitochondrial superoxide, SO_2_m, and extra-mitochondrial hydrogen peroxide, H_2_O_2i_) variables obtained at increasing concentrations of CuZnSOD as described in Figure 3A. Depicted are the logarithm of the oscillations amplitude as a function of their frequency in ΔΨ_m_ **(A)**, Succ **(B)**, SO_2_m **(C)**, and H_2_O_2i_ **(D)**. The dotted line in panel **(A)** indicates the linear trend in the double-log plot.

To better characterize mitochondrial oscillations at the edge region, we analyzed frequency (1/period) and amplitude as a function of different parametric combinations of SODs and Shunt. Within the oscillatory domain, an increase in the concentration of Cu, Zn SOD or Mn SOD (Figure 3A, compare green and black lines) or a decrease in Shunt (Figure 3A compare green and blue lines) results in lower frequency oscillations. Interestingly, different combinations of these three parameters can lead to oscillations with the same frequency (Figure 3A, dotted line), although not necessarily with the same amplitude (Figures 3B,4). For example, model simulations can reproduce the frequency of experimentally observed oscillations (~0.01 Hz, equivalent to a period of ~100 s) (Cortassa et al., 2004) for at least four distinct parametric combinations (Figure 3).

Considering the oscillations obtained under the conditions specified in Figure 3A, we examined the dependence of their amplitude vs. frequency. A double-log plot revealed an inverse relationship of amplitude vs. frequency (from >0.01 Hz) in oscillations of energetic (ΔΨ_m_, succinate) (Figures 4A,B) and redox (O^−^_2_, H_2_O_2_) (Figures 4C,D) variables obtained at different Cu, Zn SOD concentrations. According to this inverse relationship, an increase in the frequency (corresponding to a decrease in CuZnSOD concentration shown in Figure 3A) results in a decrease in the amplitude of the oscillations.

**Figure 5:**
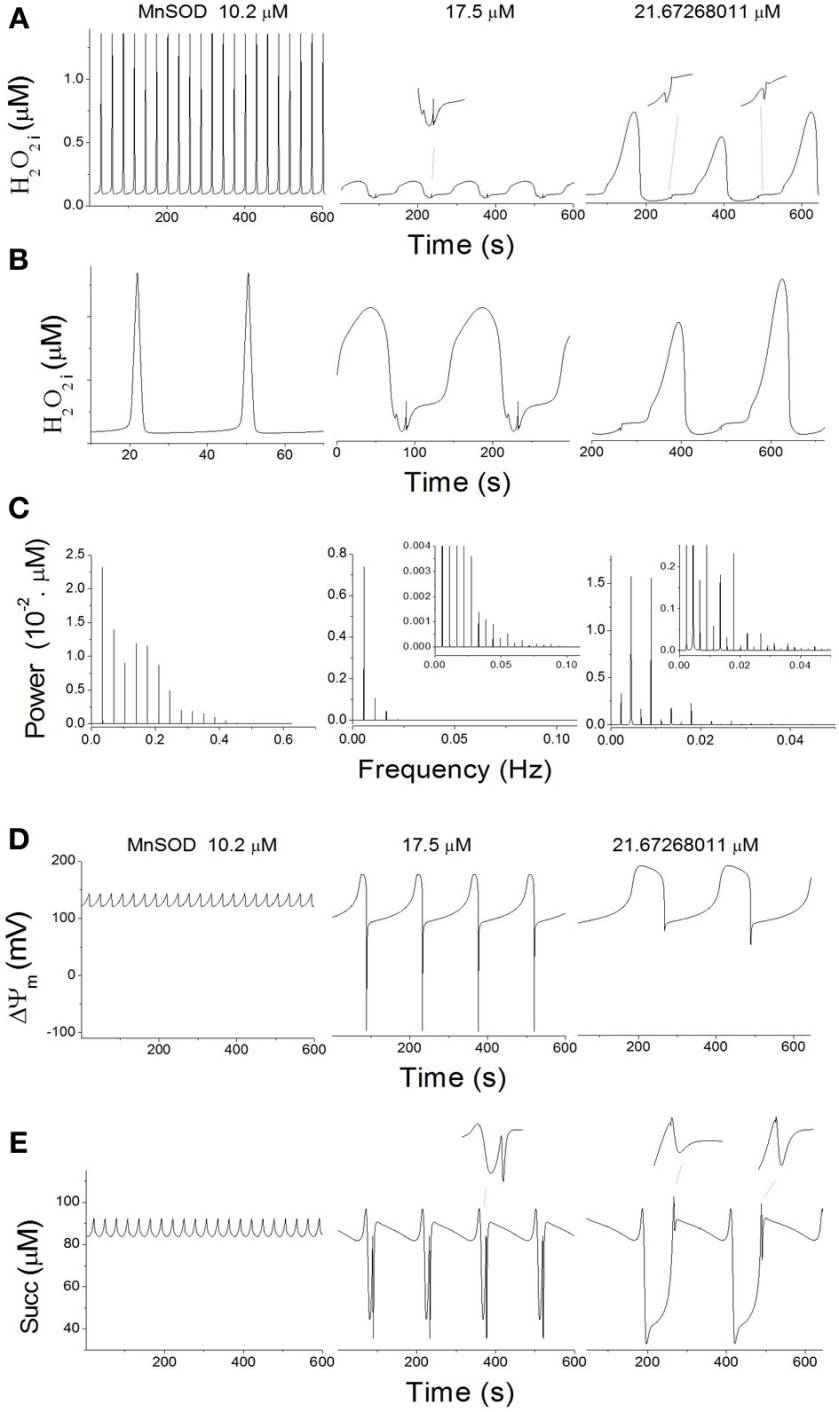
**Complex mitochondrial oscillatory dynamics**. Oscillatory dynamics in H_2_O_2i_ **(A)**, ΔΨ_m_ **(D)**, and Succ **(E)** at increasing concentrations of MnSOD at constant 9.7 μM CuZnSOD and 4% Shunt (see dotted lines in Figure 3A). **(B)** Depicted are the time series from panel **(A)** after magnification and rescaling to highlight the increase in complexity of the oscillatory waveforms. **(C)** Power spectral analysis of the time series from panel **(A)**. **(D)** Power spectral analysis of the time series from panel **(A)**, performed with a time series of 1.6 × 10^4^ s at a constant sampling interval of 1 ms.

Under oxidative stress (Shunt = 4%), increasing Mn SOD at low Cu, Zn SOD results not only in changes in frequency and amplitude, but also in the complexity of the oscillatory waveform (Figure 5). The shape of the oscillations in H_2_O_2i_ concentration shifted from a spike- to a sinusoidal-like wave form (Figures 5A,B). When the oscillatory signal corresponding to 10.2 μM MnSOD was analyzed by power spectral analysis, a high sharp peak in the frequency domain was observed at ~0.035 Hz, followed by harmonics of slightly lower values (Figure 5C). Mathematically, this time series shows similarities with a Dirac comb (also called spike train) (Kanasewich, 1981) that reflects the appearance of sharp spikes at 29 s intervals in H_2_O_2i_ concentration (Figure 5A).

At 17 μM MnSOD, three low-frequency components (~0.0055, 0.011, and 0.0165 Hz) of decreasing power can be observed in the frequency domain (Figure 5C); the ~0.0055 Hz frequency corresponds to the predominant waveform with a period of 182s (Figure 5A). Other harmonic frequencies may contribute to the complexity of the waveform (Figure 5C, inset).

At ~21.7μM MnSOD, a first lower spike at 0.00225 Hz is followed by two major spectral components of lower (~0.00444 Hz) and higher (~0.00894 Hz) frequencies (Figure 5C), equivalent to periods of ~444, 225, and 112 s, respectively. These first two spectral components clearly reflect the period doubling process, while the period of 112s marks the appearance of smaller intermediate peaks (Figure 5A). Overall, per cycle of 444s, two large and two small peaks are observed corresponding to H_2_O_2i_ concentration values of 0.535, 0.068, 0.729, and 0.077 μM. The complexity of the waveform is further underscored by a large number of contributing harmonic frequencies of different magnitudes (Figure 5C, inset).

Oscillations in ΔΨ_m_(Figure 5D) and succinate (Figure 5E) also show progressively complex waveforms for increasing concentrations of MnSOD (Figure 5D). However, the waveform complexity of ΔΨ_m_ (Figure 5D) is lower than in H_2_O_2i_ (Figure 5A) and succinate (Figure 5E).

**Figure 6:**
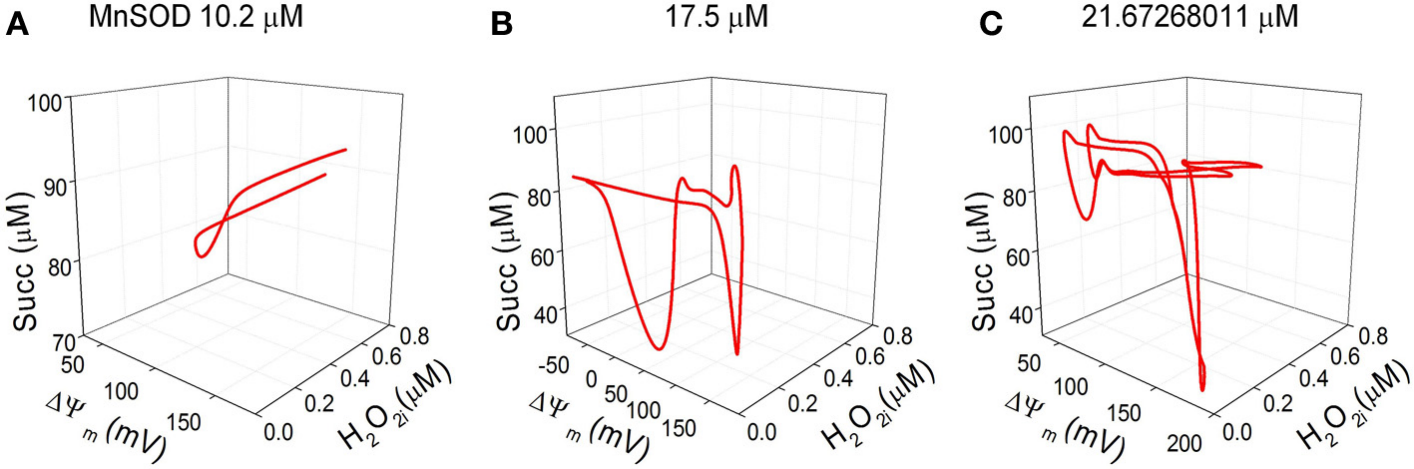
**Three-dimensional (3D) phase space projections of energetic and redox state variables**. 3D phase space plots depicting the interrelationship between the dynamic trajectories of the state variables H_2_O_2i_, ΔΨ_m_ and succinate for MnSOD **(A)** 10.2, **(B)**17, and **(C)** ~21.7 μM. The attractors described in the 3D phase plots correspond to the same time series shown Figures 5A,D,E.

Phase space 3D projections of the state variables H_2_O_2i_, ΔΨ_m_ and succinate demonstrate their complex dynamic interrelationships. This can be judged by the shape of the attractors (Figure 6) that exhibit the highest intricacy at the maximal concentration of MnSOD tested (Figure 6C; see also the corresponding time series in Figures 5A,D,E).

Overall, the results obtained indicate that the complexity of the oscillations waveform is enhanced as a function of increasing oxidative stress conditions.

## Discussion

The main contribution of the present work is to show that the interplay of Cu, Zn SOD (SOD1) and Mn SOD (SOD2) activities determines the appearance of complex oscillations in mitochondrial dynamics. The complexity of the oscillations is characterized by at least more than one period, amplitude and/or type of waveform (e.g., spikes, sinusoid) and increased at high ROS production while the antioxidant capacity of the periplasmic-cytoplasmic compartments remained low. Under these conditions, the combination of SOD activities in both compartments defines an “edge” region that delimits normal from pathological mitochondrial states. Complex oscillations occur within the “edge” region, presenting a distinct number of amplitudes and frequencies that appear inversely related when represented in a double log plot (Figure 4).

Of note is that none of the other parameters from our model, apart from the three studied herein, were capable of eliciting oscillatory behavior. The range of parametric variation in “Shunt” and SOD concentrations utilized in the present work are within realistic ranges. “Shunt” was varied between 0.1 and 8% and the extent of electron diversion from the respiratory chain to produce ROS reportedly ranged from 0.15 to 11% of the O_2_ consumption flux (Boveris et al., 1972; Chance et al., 1979; St-Pierre et al., 2002; Hoffman and Brookes, 2009; Aon et al., 2012), depending on species and whether mitochondria are in respiratory states 4 [zero ADP] or 3 [ADP present] (Aon et al., 2012). As for the SOD concentrations, values reported are ~0.5 μM (McAdam et al., 1977; Chance et al., 1979; Hsu et al., 1996) and we used a range of concentrations between 0.009 and 0.16 μM for Cu, Zn SOD, and 0.1 and 65 μM for Mn SOD.

In the model, it is noteworthy that the transition between steady state and oscillatory dynamics is shown to occur in a parametric domain of ROS production and scavenging compatible with values found in nature. The ME-R model with antioxidant arrays in both compartments renders O^−^_2_, and H_2_O_2_ levels in the pM to nM range (Kembro et al., 2013). Thus, the oscillatory release of H_2_O_2_ from the mitochondrial compartment in the ME-R model possesses modulatory potential in both amplitude and frequency that, under critical oxidant stress, may function as a signal for redox-modulated processes (Aon, 2013; Cortassa and Aon, 2013).

A relevant example of redox signaling is represented by the regulation of protein activity and the transduction of signals to downstream proteins through oxidative modification of reactive cysteine residues by ROS, and more specifically H_2_O_2_ (Finkel, 2000; Paulsen and Carroll, 2010; Aon, 2013; Kembro et al., 2014). A recent example of redox signaling involving H_2_O_2_ was shown in the synchronization of thousands of bacterial colonies (Prindle et al., 2012). There, two synergistic modes of communication appear to be involved: quorum sensing (correlated to population density within a colony) that can produce N-acyl homoserine lactones as signaling molecules, and redox signaling (H_2_O_2_ vapor) between colonies (Prindle et al., 2012). The stronger, yet short-range, quorum sensing appears to be necessary to coherently synchronize the weaker, yet long-range, redox signaling. Local and long-range effects of signaling mechanisms, across organelles within cells and cells within populations have also been shown in cardiac and yeast cells (Aon et al., 2007b, 2008a; Lloyd and Murray, 2007; Roussel and Lloyd, 2007). These are yet other examples showing that the mechanism of functional synchronization across temporal and length scales are universal among organisms separated by billions of years of evolution (Lloyd et al., 2012).

**Figure 7:**
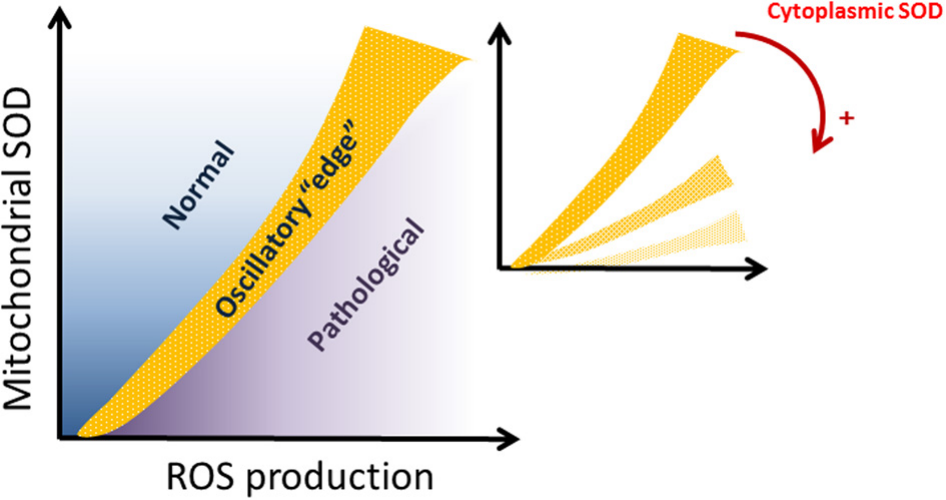
**Schematic representation of the dependence of mitochondrial dynamics on SODs compartmentalization**. The domain delimiting normal from pathological mitochondrial function (represented in light brown) corresponds to the “edge” oscillatory region. The “edge” domain is characterized by the existence of complex oscillations. The inset shows how the “edge” is displaced toward the bottom of the graph as the antioxidant capacity given by CuZn SOD in the periplasmic-cytoplasmic (extra-mitochondrial) compartment increases. Red arrow and plus sign, indicate increasing concentrations of cytoplasmic SOD.

The emergence of complex oscillatory behavior within the “edge” region, a major finding of this study, is determined by the interplay between the antioxidant powers granted by SOD1 in the extra-mitochondrial compartment and the balance of ROS production and scavenging within mitochondria (i.e., Shunt and MnSOD, respectively). Given the exchange of ROS species between compartments, the compartmentalization of SODs and their relative activities play a significant role in defining the extent of functional vs. pathological behavior, as well as the appearance of the “edge” region between both, populated by oscillatory dynamics. This main result is shown schematically in Figure 7. Oscillations occurred in a restricted region of the parametric space defined by the SODs and ROS production in the respiratory chain (denoted with light brown in Figure 7, which is a scheme of the results displayed in Figure 2). The oscillatory domain locates at the edge between normal and pathological states of mitochondria, as a function of the two parameters from the mitochondrial compartment: MnSOD vs. ROS production (shunt). Interestingly, the oscillatory domain moves toward the bottom of the plot in Figure 7, when CuZnSOD from the extra-mitochondrial compartment increases (Figure 7, inset). This result suggests that the higher the antioxidant capacity of the periplasm-cytoplasm, the larger the parametric space compatible with functional behavior. In addition, the oscillatory domain defining the edge between normal vs. pathological is also displaced toward more restricted parametric combinations. As a result when Cu, Zn SOD concentration increases, the ability of the two compartments to tolerate higher mitochondrial ROS production is enhanced, even at low concentrations of MnSOD.

Under functional conditions, mitochondria exhibit stable steady states (Figures 2B,D,F, light blue color) that may extend to the “edge” behavioral regimen (Figures 3B,D,F, various colors). The extent and the transition to the edge are determined by the extra-mitochondrial SOD1 activity, and its interplay with SOD2 and ROS production from the mitochondrial compartment (Figures 2, 7). The functionally compatible “edge” domain exhibits conspicuous behavior. On the one hand, although the overall dynamic landscape is S-shaped it does not belong to classical bistable systems since abrupt transitions do not occur between stable and unstable states (Figure 2). Instead, a more or less gradual transition between branches of stable steady states and oscillatory ones happen. On the other hand, inside the “edge” region, the model dynamics exhibits a rich variety of bifurcation properties as revealed by the existence of several Hopf bifurcations (i.e., a signature of limit cycle, oscillatory behavior) with manifold positive eigenvalues (Figure 2).

The amplitude and frequency components of the oscillations obtained at different Cu, Zn SOD concentrations are inversely related when represented in a double log graph (Figure 4). This behavior is critically dependent on SOD activities through their impact on the balance between ROS production and ROS scavenging. This is exemplified in Figure 4, where the relationship between the rate of mitochondrial superoxide, SO_2_m, production and its dismutation by MnSOD appear to be the difference responsible for the “kinks” depicted in Figure 4C. In particular, this change in behavior of the amplitude vs. frequency relationship in the SO_2_m oscillation is given by the drastic difference that occurs at high rates of ROS production (8% Shunt) between the green and black traces in Figure 4C, where the former corresponds to lower MnSOD (10 μM) than in the latter (65 μM) (Figure 4). In low MnSOD, the SO_2_m oscillations amplitude first rises to then decrease as frequency increases (elicited by decreasing CuZnSOD concentrations; Figure 3). In principle, this deviation from a straightforward inverse relationship can be explained by a dynamic mismatch between the rates of SO_2_m production and dismutation coupled to the dependence of ROS transport between compartments on the concentration gradient of these molecules across the membrane, as accounted for by the model (Kembro et al., 2013).

The inverse amplitude vs. frequency relationship was demonstrated previously (Aon et al., 2006) and is confirmed by the present, more elaborate, ME-R model (Kembro et al., 2013). The likelihood of high-frequency, low amplitude oscillations in mitochondrial ROS and ΔΨ_m_ was predicted from a computational model of the mitochondrial oscillator (Cortassa et al., 2004) and later experimentally demonstrated in cardiomyocytes (Aon et al., 2006) and oscillating, self-synchronized, yeast cultures (Murray and Lloyd, 2007; Roussel and Lloyd, 2007; Aon et al., 2008a). Theoretical simulations indicated that the mitochondrial oscillator's period can be modulated over a wide range of time scales (Cortassa et al., 2004; Aon et al., 2006, 2008b). Although the frequency distribution is broad under normal conditions, the long-term temporal correlations of the mitochondrial network could theoretically allow a change in one time scale to be felt across the frequency range, a feasible behavior in systems exhibiting inverse power law relations (Yates, 1992; West, 1999; Aon et al., 2008c; Sasidharan et al., 2012). These results led to the idea that mitochondrial oscillations may play a role as intracellular timekeeper (Aon et al., 2007a; 2008b,c).

Through frequency and amplitude modulation oscillatory dynamics may function as a temporal-encoding signaling mechanism, and ROS-induced ROS release (Zorov et al., 2000; Aon et al., 2003; Zhou et al., 2010) act as an effective coupling and synchronizing mechanism of networked mitochondria because it can exert both local and cell-wide influence (Aon et al., 2004). The present work further adds to this picture in that the inverse relationship between the amplitude and frequency components of the oscillatory H_2_O_2_ release from mitochondria (Figure 4) includes the spatio-temporal functional interdependence between biochemical processes localized in mitochondrial matrix and extra-matrix compartments as depicted in Figures 1, 6. Specifically, the 3D phase space projection of the dynamics of H_2_O_2_ released as a function of other energetic variables (ΔΨ_m_, succinate) (Figure 6) demonstrates the dynamic-functional interrelationships between processes occurring within the same time scale (seconds). This represents a profound insight into the architectural dynamics of complex systems composed of several interrelated dynamic subsystems like the one exemplified by the M-ER model (e.g., membrane potential, SOD activity, respiration, ionic transport). Dynamically speaking, these systems can potentially switch back and forth between low (steady states as fixed point attractors, “simple” limit cycles) and high dimensional dynamic regimens (complex oscillations, chaos) consisting of many degrees of freedom, in this case through slight variations in either ROS production or ROS scavenging. This itinerant dynamic motion (Kaneko and Tsuda, 2003) may confer flexibility to favor the ubiquitous adaptability and evolvability exhibited by organisms in their evolutionary processes. By modulating SOD, cells could have evolved an adaptive compromise between relative constancy (“homeostasis”) and the flexibility required under stressful redox/energetic conditions that we have previously redefined as homeodynamics (Lloyd et al., 2001). Unicellular and multicellular organisms match the time dependencies of their internal environments with the periodicities of the external world in the circadian (24 h), ultradian (<24 h), and infradian (>24 h) domains (Lloyd and Murray, 2007; Lloyd and Rossi, 2008; Lloyd et al., 2012). Thus, understanding the mechanisms by which the dynamic elements of complex systems (e.g., biochemical reactions within networks, organelles within cells, coupled oscillators in cell populations) synchronize their function across temporal and length scales becomes a crucial biological problem.

To conclude, we have shown that duplication of antioxidant defenses in different subcellular compartments may represent a powerful strategy in the evolutionary toolkit. Using this strategy cells can control ROS levels and modulate their dynamics with signaling purpose within functionally compatible states.

### Conflict of interest statement

The authors declare that the research was conducted in the absence of any commercial or financial relationships that could be construed as a potential conflict of interest.
